# Combined purebred and crossbred genetic evaluation of Columbia, Suffolk, and crossbred lamb birth and weaning weights: systematic effects and heterogeneous variances

**DOI:** 10.1093/jas/skad410

**Published:** 2023-12-12

**Authors:** Napoleón Vargas Jurado, David R Notter, Joshua B Taylor, Daniel J Brown, Michelle R Mousel, Ronald M Lewis

**Affiliations:** Department of Animal Science, University of Nebraska–Lincoln, Lincoln, NE 68583, USA; School of Animal Sciences, Virginia Tech, Blacksburg, VA 24061, USA; USDA, ARS, Range Sheep Production Efficiency Research Unit, U.S. Sheep Experiment Station, Dubois, ID 83423, USA; AGBU, a joint venture of NSW Department of Primary Industries and University of New England, 2351 Armidale, Australia; USDA-ARS Animal Disease Research Unit, Washington State University, Pullman, WA 99164, USA; Department of Animal Science, University of Nebraska–Lincoln, Lincoln, NE 68583, USA

**Keywords:** Crossbreeding, multiple-trait evaluation, systematic effects, validation

## Abstract

Despite the benefits of crossbreeding on animal performance, genetic evaluation of sheep in the U.S. does not directly incorporate records from crossbred lambs. Crossbred animals may be raised in different environments as compared to purebreds. Systemic factors such as age of dam and birth and rearing type may, therefore, affect purebred and crossbred performance differently. Furthermore, crossbred performance may benefit from heterozygosity, and genetic and environmental variances may be heterogeneous in different breeds and their crosses. Such issues must be accounted for in a combined (purebred and crossbred) genetic evaluation. The objectives of this study were to i) determine the effect of dam age and birth type on birth weight, and dam age and birth-rearing type on weaning weight, in purebred and crossbred lambs, ii) test for heterogeneous genetic and environmental variances in those weights, and iii) assess the impact of including weights on crossbred progeny on sire estimated breeding values (**EBV**). Performance records were available on purebred Columbia and Suffolk lambs. Crossbred information was available on lambs sired by Suffolk, Columbia or Texel rams mated to Columbia, Suffolk, or crossbred ewes. A multiple-trait animal model was fitted in which weights from Columbia, Suffolk, or crossbred lambs were considered different traits. At birth, there were 4,160, 2,356, and 5,273 Columbia, Suffolk, and crossbred records, respectively, with means (SD) of 5.14 (1.04), 5.32 (1.14), and 5.43 (1.23) kg, respectively. At weaning, on average at 122 (12) d, there were 2,557, 980, and 3,876 Columbia, Suffolk, and crossbred records, respectively, with corresponding means of 39.8 (7.2), 40.3 (7.9), and 39.6 (8.0) kg. Dam age had a large positive effect on birth and weaning weight in pure and crossbred lambs. At birth, however, the predicted effect was larger in crossbred and Suffolk lambs. While an increase in a number of lambs born and reared had a strong and negative influence on birth and weaning weight, the size of the effect did not differ across-breed types. Environmental variances were similar at birth and weaning, but additive variances differed among breed types for both weights. Combining purebred and crossbred information in the evaluation not only improved predictions of genetic merit in purebred sires but also allowed for direct comparisons of sires of different breeds. Breeders thus can benefit from an additional tool for making selection decisions.

## Teaser

Dam age, and birth type or birth and rearing type, affected the weights of purebred lambs and their crosses differently. Estimates of these systematic effects, along with estimates of direct and maternal heterosis, can be used to adjust birth and weaning weights in purebred and crossbred lambs to allow their direct comparison in an across-breed genetic evaluation.

## Introduction

Crossbreeding is an important strategy to improve the efficiency of sheep production systems, especially under extensive conditions (Gootwine, 2020; de Barbieri et al., 2021). Lambs can benefit from crossbreeding directly or due to their dams also being crossbred (Murphy et al., 2018). Despite these advantages, crossbred data are not directly incorporated in the U.S. sheep genetic evaluation.

Genetic evaluation of sheep in the U.S. by the National Sheep Improvement Program accommodates several breed types including western-range, terminal, semi-prolific, and hair sheep (Notter, 1998; Vargas Jurado et al., 2022). Currently, evaluations are performed within a breed type and as no linkages exist among breeds, they are within the breed. In practice, however, commercial operations commonly use terminal-sire breeds (e.g., Suffolk) on western-range (e.g., Columbia) or semi-prolific ewes (e.g., Polypay). As such, genetic linkages or connectedness between purebred and crossbred animals exist. Consequently, this kind of information could be used in genetic evaluation to increase the number of records, perhaps generated in environments different from those of purebred animals, potentially increasing the accuracy of estimated breeding values (**EBV**) of purebred animals obtained via best linear unbiased prediction (**BLUP**).

In a joint purebred and crossbred evaluation several factors must be considered, including the effects of direct and maternal heterosis on crossbred lamb performance (Van Raden, 1992). In addition, due to breeds having different breeding and selection objectives, it is necessary to account for variability within and across breeds by fitting genetic groups (Brown et al., 2016; Vargas Jurado et al., 2022). Because crossbred animals may be raised in different environments than their purebred parents, direct comparisons may be challenging. As such, it is important to properly define contemporary groups to account for different environments and to ensure those environments are connected to ensure equitable genetic comparisons.

Systematic and environmental effects such as feeding practices, dam age, and litter size have an impact on lamb performance (Morris and Kenyon, 2004; Chniter et al., 2011). In addition, the extent to which these effects are observed in the performance of lambs may be breed dependent (Freetly and Leymaster, 2004). Among these, dam age and type of birth and rearing are frequently recorded in practice and included in routine genetic evaluations. However, heterosis and other environmental factors can also impact the extent to which such systematic effects affect lamb performance. As such, in multiple-breed evaluations including crossbred progeny, it may be necessary to tailor adjustments for the effect of dam age, and birth and rearing type, to specific breed types. Moreover, because crossbred animals may be raised in environments different from those of purebreds, bias may potentially be introduced into the prediction of breeding values. Correspondingly, the aims of this study were to i) determine the effect of dam age and birth type on birth weights, and dam age and birth-rearing type on weaning weights, in purebred and crossbred lambs, ii) test for heterogeneity of additive and residual variances in those weights, and iii) assess the impact of including the performance of crossbreds in the genetic evaluation of purebreds.

## Materials and Methods

Data used in the study were from various sheep genetics projects conducted at the USDA, Agricultural Research Service, Range Sheep Production Efficiency Research Unit, U.S. Sheep Experiment Station (**USSES**). A detailed description of the location, vegetation types and ecosystems, climate variables, and grazing systems at USSES can be found in Seefledt and McCoy (2003), Leytem and Seefeldt (2008), Taylor et al. (2009), Moffet et al. (2015), and Notter et al. (2017). All husbandry practices and related animal-care procedures were reviewed and approved by the USSES Institutional Animal Care and Use Committee.

### Purebred and crossbred records

Suffolk, Columbia, and Texel rams were acquired for use in a terminal-sire evaluation project (**TS1**) that took place between 2005 and 2007 (Leeds et al., 2012), and an additional set of Suffolk sires were obtained for a second terminal-sire evaluation (**TS2**) that occurred between 2015 and 2017 (Notter and Taylor, 2019). In TS2, beyond Suffolk, the progeny of Siremax and a paternal composite breed were compared (Vargas Jurado et al., 2022). The crossbred lambs from these two projects were out of Rambouillet, Targhee, and Polypay dams, and their records were not included in the current study. However, the purchased sires, as well as a sample of Suffolk and Columbia sires from the existing USSES flock, were used as founder sires to produce the crossbreds considered in this study (Vargas Jurado et al., 2022).

Lambs used in the analyses were sired by purebred (number of lambs) Suffolk (*n* = 3,572), Columbia (*n* = 4,543) or Texel (*n* = 591) rams, or by crossbred rams (*n* = 3,083). Crossbred rams (number of crosses) were Suffolk × Columbia (*n* = 160), Columbia × Suffolk (*n* = 84), Texel × Suffolk (*n = *150), Texel × Columbia (*n* = 310), Suffolk × Texel (*n* = 29), ½ Suffolk ¼ Texel ¼ Columbia (*n* = 204), ½ Columbia ¼ Texel ¼ Suffolk (*n* = 280), ½ Texel ¼ Suffolk ¼ Columbia (*n* = 510), ¾ Columbia ¼ Texel (*n* = 209), ⅜ Columbia ⅜ Suffolk ¼ Texel (**PC**; *n* = 1,105), ⅜ Columbia ¼ Suffolk ⅜ Texel (*n* = 28), and ⅜ Columbia ½ Suffolk ⅛ Texel (*n* = 14).

### Traits and data processing

Body weight was collected on purebred and crossbred lambs at birth (0 d of age) and weaning (122 [SD 12] d on average). Lambs missing descriptive information (i.e., sex, management group) were removed. In addition, observations more than four standard deviations from the mean were considered outliers and removed. A total of 98 and 51 records were removed at birth and weaning, respectively. After removing outliers, standardized residuals were also assessed for approximate normality using quantile-quantile plots. Summary statistics including a number of records, mean, and standard deviation for each trait and breed are presented in Table 1 (after data editing).

**Table 1.** Summary statistics for birth and weaning weight for purebred Columbia and Suffolk lambs, and for crossbred lambs (after data editing)

Trait	Breed	Number of records	Mean, kg	SD, kg	Age, d (SD)
Birth	Columbia	4,160	5.14	1.04	0.0
	Suffolk	2,356	5.32	1.14	0.0
	Crossbred	5,273	5.43	1.23	0.0
Weaning	Columbia	2,557	39.8	7.15	123 (11)
	Suffolk	980	40.3	7.94	124 (12)
	Crossbred	3,876	39.6	7.95	121 (13)

### Animal management

Management practices at USSES reflected those common in extensive rangeland sheep production systems in the western regions of the U.S. Feeding and management during breeding, late gestation, and lambing were similar for Columbia, Suffolk, and crossbred ewes. However, management in mid-gestation and during the pre-weaning and post-weaning periods differed among dam breeds.

Lambs were born in early March through mid-April. Columbia and Suffolk ram lambs with conformational defects and those not intended to be used for breeding (based on their dam’s previous performance) were castrated at birth. Castrated lambs included all male lambs in TS1 and TS2. Before leaving the lambing area, lambs were assigned to a grazing management group (weaning band) based on their dam’s breed (Vargas Jurado et al., 2022). Ram lambs were weaned in July while ewe lambs and wethers were weaned in August and September. Lambs retained as replacements were managed with all breed replacements for the general flock with ewe lambs kept separate from ram lambs.

Columbia ewes and their lambs (purebred or crossbred) were managed on sagebrush steppe in spring (early May through July) and herded in summer (mid-August/early September) in subalpine forest. After weaning their lambs in August/September, ewes were maintained on sagebrush steppe until breeding when they were transitioned to a feedlot for single-sire mating for approximately 35 d. Suffolk or Suffolk crossbred sires from the USSES flock were then used as “clean-up” rams for an additional breeding cycle. Prior to 2014, after breeding, Columbia ewes were maintained on sagebrush steppe through early/mid-January and then transitioned to a feedlot for 50 to 65 d until lambing. After 2014, however, Columbia ewes grazed a combination of sagebrush steppe and (or) crop residue (e.g., alfalfa), depending on annual availability, until late December/early January and then transitioned to a feedlot for 60 to 75 d until lambing.

Suffolk ewes and their purebred and crossbred lambs were managed on improved, sub-irrigated, fenced pastures in the summer until weaning in early fall. Suffolk ewes grazed sagebrush steppe and (or) crop residue with ewes of the other breeds after weaning but were moved to a feedlot environment earlier than ewes of the other breeds depending on the severity of winter conditions. Prior to 2014, crossbred ewes of up to 50% Suffolk breeding were managed alongside Columbia ewes during summer. However, after 2014 all Suffolk, Columbia, and terminal-sire crossbred (including PC) ewes were assigned to the same management group and grazed together on the pastures previously described for the Suffolk ewes. This was done to minimize potential bias when comparing the performance of Suffolk ewes with the performance of other terminal-sire crossbred types.

### Genetic groups

Due to the different breeds used in the current study, and to account for potential differences in the genetic merit of individuals in the pedigree, 7 genetic groups were defined consisting of 3 Columbia groups, 3 Suffolk groups, and 1 Texel group. For the Columbia and Suffolk breeds, genetic groups were determined based on the year of birth (or purchase) of sires and dams. A more detailed description of the genetic groupings can be found in Vargas Jurado et al. (2022).

### Multiple-trait model

Separately for birth and weaning weights, the performance of purebred Columbia, Suffolk, and crossbred lambs was considered as different traits (indexed by 1, 2, and 3). Hereinafter we use the term “breed type” to denote both Columbia and Suffolk purebred as well as crossbred lambs. For each weight, a tri-variate model was fitted in ASReml 4.0 (Gilmour et al., 2015)


$$y=\text{X}\beta +{\text{Z}}_{\text{D}}{\text{a}}_{\text{D}}+{\text{Z}}_{\text{M}}{\text{a}}_{\text{M}}+{\text{Z}}_{\text{D}}{\text{Q}}_{\text{g}}+{\text{W}}_{\text{c}}+\text{e}$$ (1)

where $y\prime =[y_{1}^{\prime },y_{2}^{\prime },y_{3}^{\prime }]$ was the vector of weight records, $\beta =[\beta_{1},\beta_{2},\beta_{3}]$ was the vector of fixed effects, $a_{\text{D}}=[a_{{\text{D}}_{1}},a_{{\text{D}}_{2}},a_{{\text{D}}_{3}}]$ and $a_{\text{M}}=[a_{{\text{M}}_{1}},a_{{\text{M}}_{2}},a_{{\text{M}}_{3}}]$ were the vectors of direct and maternal additive effects, respectively, $g=[g_{1},g_{2},g_{3}]$ was the vector of random genetic group effect, $c=[c_{1},c_{2},c_{3}]$ was the vector of maternal uncorrelated environmental effects, and $e=[e_{1},e_{2},e_{3}]$ was the vector of residuals. Furthermore, $X=diag(X_{1},X_{2},X_{3})$, $Z_{\text{D}}=diag(Z_{{\text{D}}_{1}},Z_{{\text{D}}_{2}},Z_{{\text{D}}_{3}})$, $Z_{\text{M}}=diag(Z_{{\text{M}}_{1}},Z_{{\text{M}}_{2}},Z_{{\text{M}}_{3}})$, and $W=diag(W_{1},W_{2},W_{3})$ were design matrices for fixed, direct and maternal additive, and maternal uncorrelated environmental effects, respectively. The matrix **Q** contained genetic group fractions for all individuals in the pedigree, and diag(⋅) referred to a block diagonal matrix.

#### Fixed effects

The selection of systematic (fixed) effects defining variation in birth and weaning weight was performed using a linear model in R (R Core Team, 2021). Residual error was included as a random effect. Only those main and interaction effects that defined significant variation (*P* < 0.05) in a trait were retained in the final model.

Fixed effects depended on the trait. At birth, they included birth type (single, twin, and triplet or more lambs born), dam age (1, …, 7 + yr of age), birth type by dam age interaction, and, as a covariate, lambing day (day of the year). At weaning effects included birth and rearing category (single and single; twin+ and single; twin+ and twin; and twin+ and twin+), dam age, dam age by birth and rearing category, and, as a covariate, age at weaning. The contemporary group was defined as the combination of sex, year of birth, management group (up to three weaning bands within each year), date of weight measurement, and birth slice. Birth slice was defined as sequential 35-d intervals from the birth of the first lamb in a lambing season.

Differences in the distribution of dam age, birth type, and birth and rearing type among breed types at birth and weaning were determined using a contingency table analysis in R (Chi square test). For the crossbred lambs (crossbred traits), in addition to the systematic effects described above, direct and maternal heterotic effects were included as continuous covariates. The expected breed heterozygosity was calculated as


$$\begin{array}{l} Heterozygosity=1-\underset{i=1}{\overset{3}{\sum }}\;f_{ij}^{S}f_{ij}^{D} \end{array}$$

where $f_{ij}^{S}$ and $f_{ij}^{D}$ (*i* = 1, 2, 3 for Columbia, Suffolk, and Texel) were the expected fractions of breed i for the sire and dam, respectively, of individual *j*. The expected breed fractions were calculated in Van Vleck (1997) and assumed to be directly proportional to the fraction of maximum heterosis that was expected for each animal.

#### Random effects

A base model including random effects was adopted from Vargas Jurado et al. (2022). Briefly, log-likelihood ratio tests were used to determine the effects included in the final model. The models tested included direct and maternal additive effects and an additional uncorrelated maternal environmental effect. Covariance among breed types for direct and maternal additive effects was also tested. Based on the log-likelihood ratio values for different models (results not shown), the final model for birth weight included direct and maternal additive effects, and an uncorrelated maternal environmental effect. At weaning, the model included a direct additive effect and an uncorrelated maternal environmental effect. In the current study, performance records corresponding to each breed type were considered as a different trait, such that a given ewe might be the dam of purebred and crossbred lambs across different lambing events. Therefore, the uncorrelated maternal permanent environmental effect fitted at birth and weaning could not be further partitioned into maternal permanent and maternal temporary environmental effects as was the case in Vargas Jurado et al. (2022). Similarly, additive maternal effects were not included at weaning as their effects were not significant based on model fit (Vargas Jurado et al., 2022).

Direct additive and, where fitted, maternal additive effects were assumed to be correlated across-breed types. However, these additive covariances between Columbia and Suffolk were set to zero because they were deemed not estimable during preliminary model testing. Genetic group, maternal uncorrelated environmental, and residual effects were assumed to be independent among breed types (i.e., diagonal variance structure). The inclusion of maternal genetic group effects was considered during the model selection, but it was not possible to include them (i.e., not estimable).

Homogeneity of variances (residual and additive) across-breed types was tested using a Bartlett’s and a log-likelihood ratio test (0.05 significance level). For the log-likelihood ratio test, model (1) was fitted with (i) either a diagonal residual variance structure, that is where the residual variance was estimated within a breed (i.e., $\sigma_{e_{1}}^{2}$, $\sigma_{e_{2}}^{2}$, and $\sigma_{e_{3}}^{2}$ for Columbia, Suffolk, and crossbred lambs, respectively) or a common residual variance (i.e., $\sigma_{e}^{2}$), and (ii) either a diagonal additive variance structure ($\sigma_{A_{j_{1}}}^{2}$, $\sigma_{A_{j_{2}}}^{2}$, and $\sigma_{A_{j_{3}}}^{2}$ for Columbia, Suffolk, and crossbred lambs, respectively), or a common additive variance ($\sigma_{A_{j}}^{2}$) where *j* = *D, M* reflected direct and maternal additive effects at birth or direct additive at weaning. Only one of the proposed scenarios (residual, direct, and maternal) was tested at a time such that the corresponding likelihood ratio test had two degrees of freedom. The direct additive and maternal effects were also tested to determine whether the covariance among breed types was nonzero. This was achieved by including one covariance component at a time (pairs of breeds), which resulted in a likelihood ratio test with one degree of freedom.

Based on the likelihood ratio and Bartlett’s test, it was assumed that $Var[a_{\text{D}}]=G_{\text{D}}⊗A$, and $Var\;[\;a_{\text{M}}\;]\;=G_{\text{M}}⊗A$, where **G***_D_* and **G***_M_* were unstructured (co)variance matrices, **A** was the numerator relationship matrix, and ⊗ was the Kronecker product. Also, it was assumed that genetic group effects, uncorrelated maternal environmental, and residual effects had a diagonal variance structure (i.e., were uncorrelated across-breed types). Variance components were estimated using the average information Restricted Maximum Likelihood algorithm.

### Connectedness

Connectedness, a measure of genetic relatedness, was calculated among breed types based on prediction error correlations (Lewis et al., 1999; Kuehn et al., 2007). To obtain this statistic, prediction error covariances among EBV (direct additive effects) for each trait were obtained. The model fitted included contemporary group, dam age, and either birth type (for birth weight) or birth-rearing type (for weaning weight). For the analysis of connectedness, the contemporary group was defined as nested within a breed such that it mimicked the specification used in the multiple-trait analyses.

### Validation

**Figure 1.**
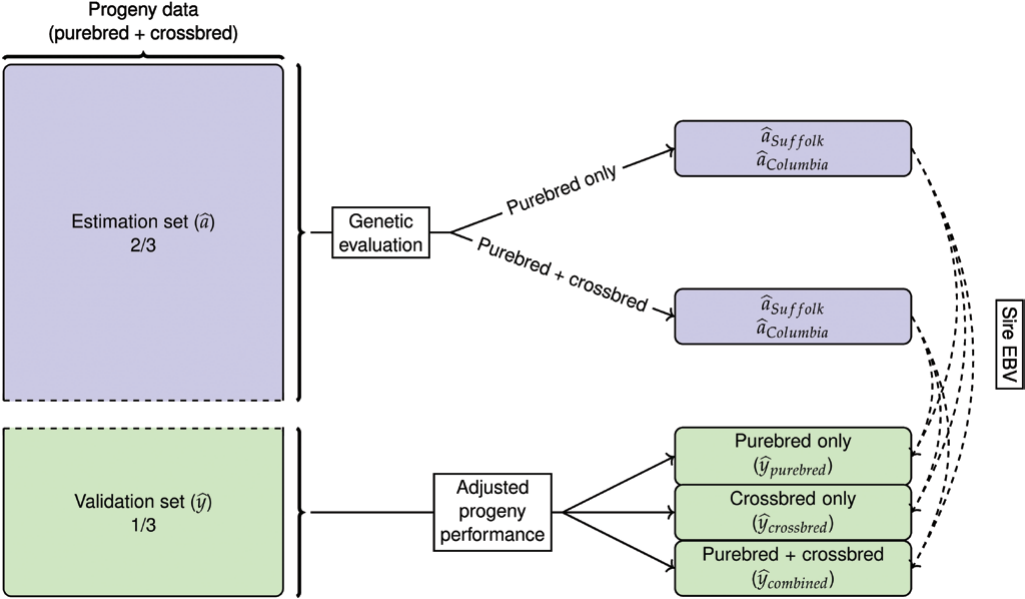
Cross-validation design where $\hat{a}$ represent EBV of the progeny of the purebred sires and $\hat{y}$ represent the adjusted progeny performance. (Adjusted progeny performance fitted as dependent variable in a linear model, with the contemporary group, dam age, birth type [for birth weight] or birth and rearing type and age at measurement [for weaning weight] as fixed effects, and sire EBV and heterosis [for crossbred progeny] as continuous covariates, and a random residual.) Dashed-line arrows show linear models where sire EBV was included as continuous covariates.

The impact of including crossbred records on the EBV of purebred Columbia and Suffolk sires was assessed using cross-validation (Figure 1) which was replicated five times. Sires of each breed were selected at random and their progeny records at birth and weaning were removed (validation set) such that they corresponded to approximately 1/3 of the data while the remaining records (estimation set) corresponded roughly to 2/3 of the total data. Using the estimation set, purebred sire EBV was estimated through a i) purebred-only evaluation where only purebred progeny records were used, or a ii) combined (joint) evaluation where both crossbred and purebred progeny records were used. A homogeneous residual variance model was assumed for the prediction of EBV. Regression coefficients were obtained by fitting a linear model in which progeny records from the validation set were used as the dependent variable, with the contemporary group, dam age, birth type (for birth weight) or birth and rearing type and age at measurement (for weaning weight) as fixed effects, and sire EBV and heterosis (for crossbred progeny) as continuous covariates, and a random residual. Progeny records used were further divided into i) purebred only, ii) crossbred only, and iii) purebred and crossbred records combined. As such, six scenarios for validation were possible at birth and weaning.

In addition to the six regression scenarios mentioned above, the overall fit of the model (*R*^2^) and changes to the accuracy of purebred sire EBV were determined for the different validation scenarios. Accuracy of sire EBV was calculated as (Gilmour et al, 2015)


$$\begin{array}{l} \sqrt{1-\frac{s_{i}^{2}}{\left(1+f_{i}\right)\sigma_{A_{D}}^{2}}} \end{array}$$

where $s_{i}$ was the reported standard error for the EBV of sire *i*, $f_{i}$ was the inbreeding coefficient of sire *i*, and $\sigma_{A_{D}}^{2}$ was the direct additive variance for a given breed type and trait.

## Results

### Heterosis

For crossbred lambs at birth, direct and maternal estimates of heterosis were 0.41 ± 0.19 and 0.26 ± 0.09, respectively. These represented 7.59 ± 3.51 and 4.81 ± 1.66 % of birth weight (of crossbred lambs), respectively. Similarly, at weaning direct and maternal heterosis estimates were 4.38 ± 1.40 and 1.77 ± 0.59, respectively. These values represented 11.06 ± 3.53 and 4.47 ± 1.49 % of crossbred weaning weight, respectively.

### Connectedness among and within contemporary groups

Based on threshold values defined by Kuehn et al. (2008), the extent to which EBV could be fairly compared across purebreds was comparatively weak, while that between purebreds and crossbreds was strong (Table 2). At birth and weaning, the minimum relatedness was observed between the Texel and Suffolk breeds with connectedness correlations of 0.09 and 0.05, respectively. The maximum observed was between the crossbred and Columbia breed with connectedness correlations of 0.58 and 0.55, respectively.

**Table 2.** Connectedness (genetic relatedness) correlation coefficient (100×) among breeds including crossbred individuals at birth (lower triangle) and weaning (upper triangle)

Breed	Columbia	Crossbred	Suffolk	Texel
Columbia	—	55.2	8.8	6.7
Crossbred	57.7	—	50.1	41.3
Suffolk	13.5	50.1	—	5.3
Texel	9.0	44.5	8.5	—

### Dam age effect

**Figure 2.**
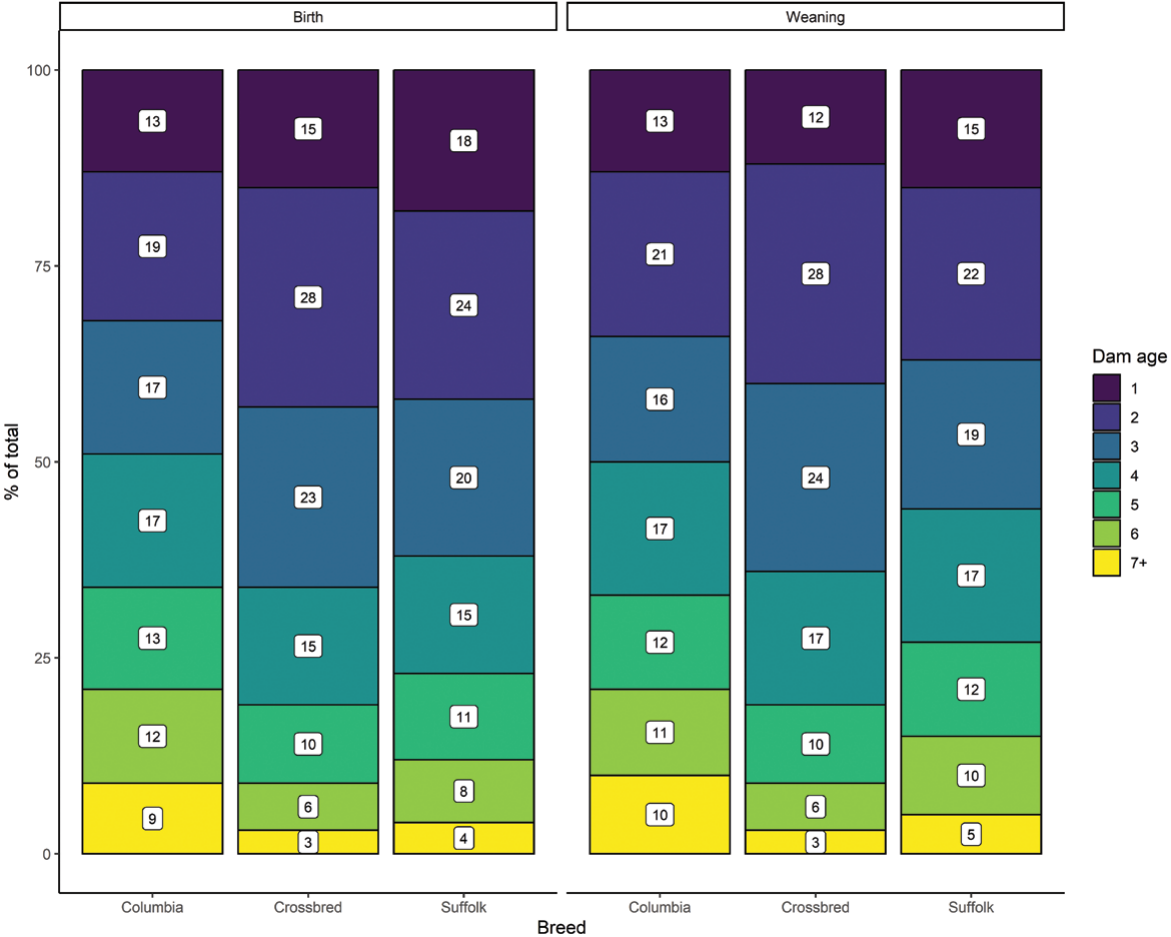
Dam age (yr) distribution (proportion of each category [%] of the total shown in white squares) at birth and weaning for purebred Columbia, Suffolk, and crossbred lambs.

The distribution of dam age (i.e., the proportion of lambs born from dams of a given age) at birth and weaning is presented in Figure 2. The number of lambs in each category differed among purebred and crossbred animals (*P* < 0.001). At birth, the (cumulative) proportion of lambs born from 1-yr-old to 3-yr-old crossbred or Suffolk ewes (0.66 or 0.62) was roughly the same as lambs born from 1-yr-old to 4-yr-old Columbia ewes (0.66). Consequently, the proportion of Columbia lambs born from 4-yr-old ewes to 7+-yr-old ewes (0.34) roughly corresponded to the proportion of crossbred and Suffolk lambs born from 3-yr-old ewes to 7+-yr-old ewes (0.34 or 0.38). A similar trend was observed at weaning for Columbia and crossbred lambs but less so for Suffolk lambs.

**Figure 3.**
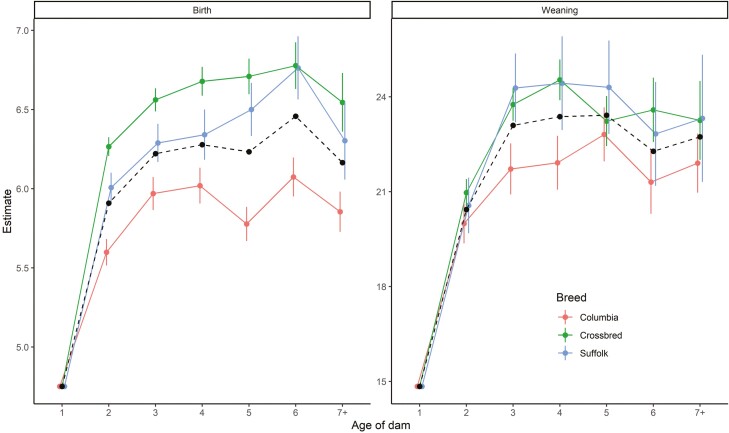
Dam age effect at birth and weaning for purebred Columbia, Suffolk, and crossbred lambs. Dashed black lines and circles show the average of the purebred dam age effect weighted by the breed composition of a lamb.

Estimates of the marginal effect of dam age at birth and weaning on weight for the Columbia, Suffolk, and crossbred lambs are shown in Figure 3. The predicted effect of dam age was quadratic (*P* < 0.05) for all breed types and for both traits. The largest predicted effect in general was observed at 6 and 4 yr of age for birth and weaning, respectively, while the smallest predicted effect was observed at 1 yr of age. At birth, the effect of dam age differed across-breed types with the largest effect observed on crossbred lambs and the lowest on purebred Columbia lambs. At weaning, on the other hand, dam age effect was different only for the Columbia breed at 3 and 4 yr of age where it was lower than that of Suffolk and crossbreds.

### Birth type and birth-rearing type

**Figure 4.**
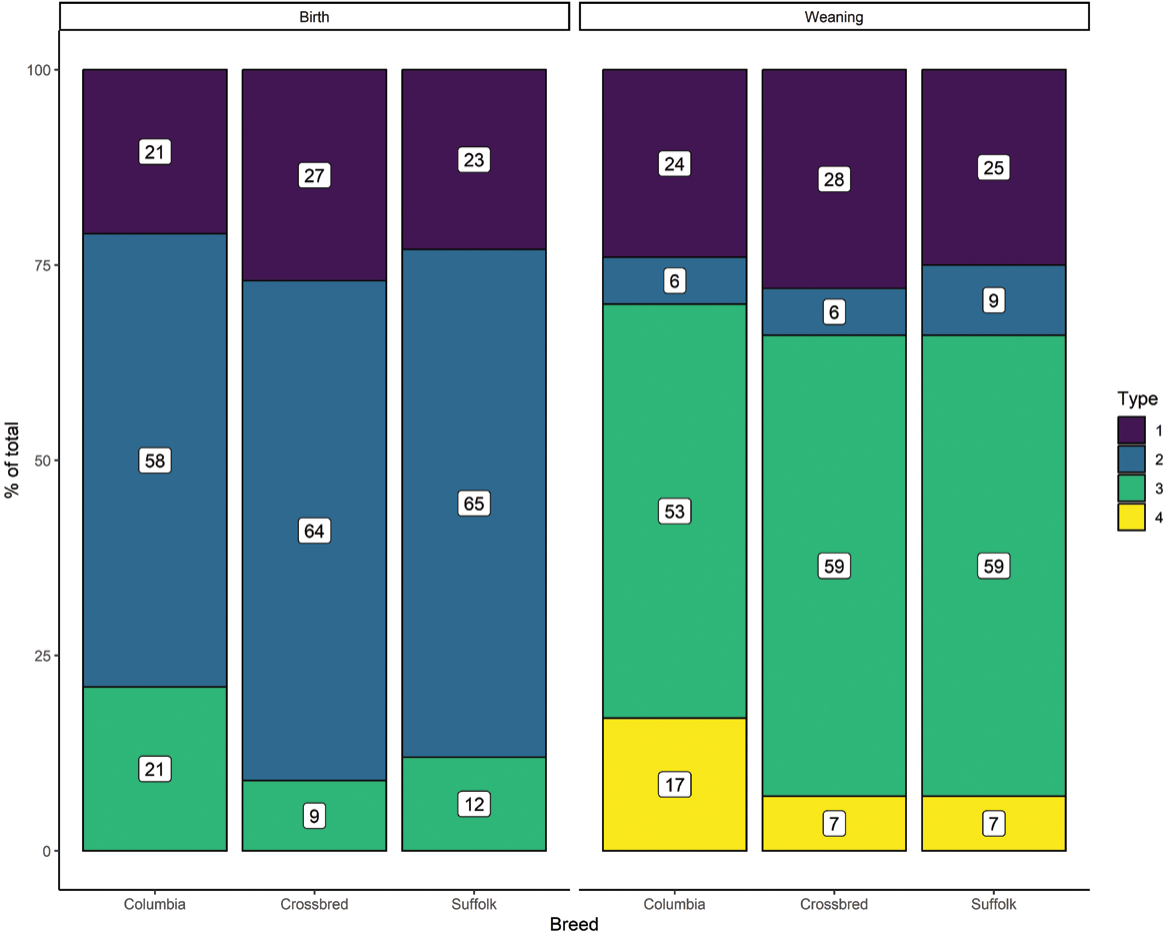
Distribution (proportion of each category [%] of the total shown in white squares) of birth type at birth (1 for single, 2 for twins, and 3 for triplets+) and birth-rearing type at weaning (1 for single and single, 2 for twin+ and single, 3 for twin+ and twin and 4 for twin+ and twin+) for purebred Columbia, Suffolk, and crossbred lambs.

As with dam age, the distribution of birth type and birth-rearing type (i.e., the proportion of lambs assigned to a given category) at birth and weaning, respectively, differed among breed types (*P* < 0.001; Figure 4). At birth, the proportion of lambs born as singles was similar for Columbia and Suffolk lambs, but slightly higher (1.28 times) for crossbred lambs. On the other hand, a smaller proportion of Columbia lambs were born as twins compared to crossbreds and Suffolk lambs. However, the proportion of Columbia lambs born as triplets+ was 2.33 and 1.75 times those of crossbred and Suffolk lambs, respectively.

At weaning, the cumulative proportion of lambs born as twins or singles, and raised as singles, was similar for all three breed types. The proportion of Columbia lambs born as twins+ and raised as twins were similar for crossbred and Suffolk lambs but lower (0.90 times) for Columbias. On the other hand, the proportion of Columbia lambs born and raised as twins+ was about 2.43 times that of crossbred and Suffolk lambs.

**Figure 5.**
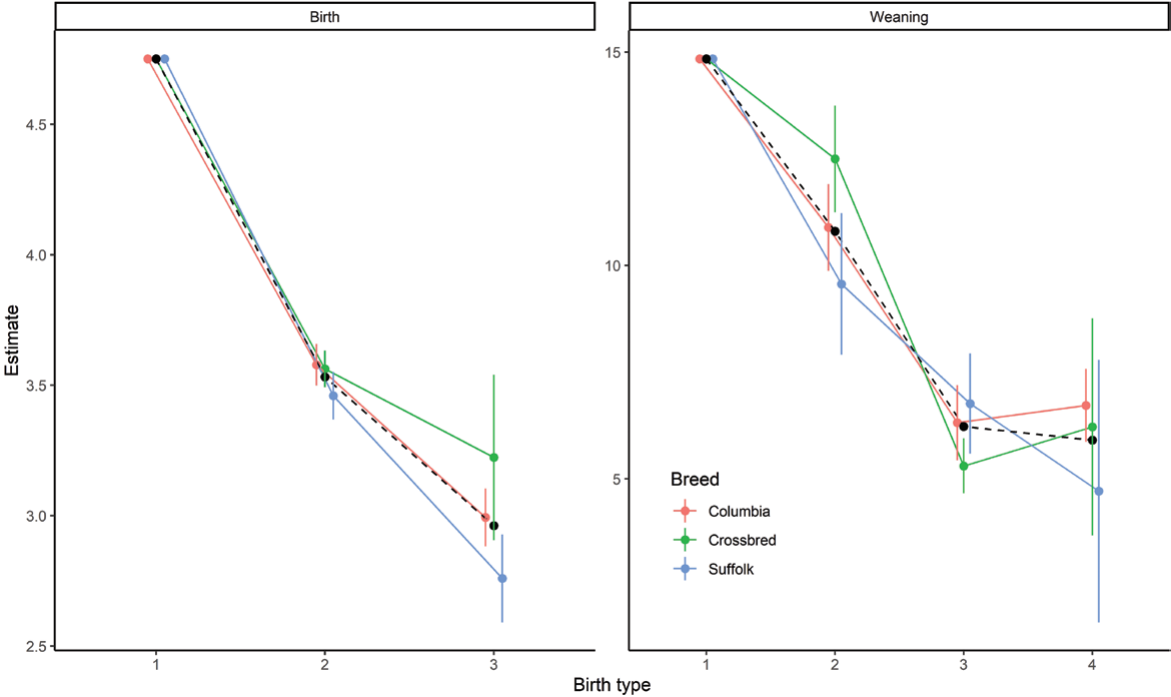
Birth type effect at birth (1 for single, 2 for twins, 3 for triplets+) and birth-rearing type effect at weaning (1 for single and single, 2 for twin+ and single, 3 for twin+ and twin and 4 for twin+ and twin+) for purebred Columbia, Suffolk, and crossbred lambs. Dashed black lines and circles show the average of the purebred birth type (or birth-rearing type at weaning) effect weighted by the breed composition of a lamb.

Estimates of the marginal effect of birth type at birth and birth-rearing type at weaning are shown in Figure 5. Birth and birth-rearing types had strong and linear negative effects (*P* < 0.05) on weight at birth and weaning, respectively. Contrary to dam age, the effects of birth and birth-rearing type on weight did not differ across-breed types.

### Variance components

Based on Bartlett’s test, variances differed among breed types. Such was the case for additive (direct and maternal) and residual variances at birth (*P* < 0.01) and at weaning (*P* < 0.001). However, based on a log-likelihood ratio test, direct and maternal additive variances differed among breeds (*P* < 0.05), but residual variances were not different (*P* > 0.10). This was the case at birth and weaning. In addition, because additive covariances (direct and maternal) among breed types were found to be significant in the model testing phase, final estimates of variance components were based on the model where direct and maternal additive variances were allowed to differ for each breed. Furthermore, nonzero direct additive covariances among breed types (except between Columbia and Suffolk) were found at birth and weaning, while nonzero maternal additive covariances between breed types (except between Suffolk and Columbia) were found only at birth. However, because residual variances were found to differ among breed types using Bartlett’s test, estimates of variance components for the homogeneous and heterogeneous residual variance cases are presented in Table 3.

**Table 3.** Estimates of phenotypic ($\sigma_{p}^{2}$), direct $\left(\sigma_{A_{D}}^{2}\right)$ and maternal $\left(\sigma_{A_{M}}^{2}\right)$ additive, and uncorrelated maternal environmental ($\sigma_{c}^{2}$) variance, direct ($h_{D}^{2}$) and maternal ($h_{M}^{2}$) heritability, and the proportion of variance accounted for by uncorrelated maternal environmental variance ($c^{2}$), for weight (kg) in Columbia, Suffolk, and crossbred lambs at birth and weaning fitting a model with heterogeneous or homogeneous residual variance. Standard errors of estimates in parentheses

Trait	Model	Component		Breed	
			Columbia	Suffolk	Crossbred
Birth	Heterogeneous	$\sigma_{p}^{2}$	0.78 (0.02)	0.86 (0.04)	0.85 (0.02)
		$\sigma_{A_{D}}^{2}$	0.11 (0.03)	0.21 (0.05)	0.16 (0.03)
		$\sigma_{A_{M}}^{2}$	0.19 (0.04)	0.21 (0.05)	0.17 (0.03)
		$\sigma_{c}^{2}$	0.07 (0.03)	0.03 (0.03)	0.06 (0.02)
		$\sigma_{e}^{2}$	0.41 (0.02)	0.41 (0.03)	0.46 (0.03)
		$h_{D}^{2}$	0.14 (0.03)	0.24 (0.05)	0.19 (0.03)
		$h_{M}^{2}$	0.24 (0.05)	0.25 (0.04)	0.20 (0.03)
		$c^{2}$	0.09 (0.03)	0.04 (0.04)	0.07 (0.03)
	Homogeneous^1^	$\sigma_{p}^{2}$	0.77 (0.02)	0.85 (0.04)	0.84 (0.02)
		$\sigma_{A_{D}}^{2}$	0.11 (0.03)	0.20 (0.05)	0.16 (0.03)
		$\sigma_{A_{M}}^{2}$	0.18 (0.04)	0.20 (0.05)	0.18 (0.03)
		$\sigma_{c}^{2}$	0.05 (0.03)	0.02 (0.03)	0.07 (0.02)
		$h_{D}^{2}$	0.14 (0.02)	0.24 (0.03)	0.19 (0.03)
		$h_{M}^{2}$	0.23 (0.04)	0.34 (0.05)	0.21 (0.03)
		$c^{2}$	0.06 (0.03)	0.02 (0.04)	0.08 (0.03)
Weaning	Heterogeneous	$\sigma_{p}^{2}$	27.37 (1.11)	28.14 (1.41)	24.75 (0.95)
		$\sigma_{A_{D}}^{2}$	4.57 (1.23)	3.42 (1.00)	4.24 (1.42)
		$\sigma_{c}^{2}$	1.55 (0.57)	3.08 (1.30)	3.02 (0.58)
		$\sigma_{e}^{2}$	21.25 (0.95)	21.64 (1.10)	17.49 (0.80)
		$h_{D}^{2}$	0.17 (0.07)	0.12 (0.05)	0.17 (0.04)
		$c^{2}$	0.06 (0.02)	0.11 (0.05)	0.12 (0.05)
	Homogeneous^2^	$\sigma_{p}^{2}$	25.96 (1.10)	28.02 (1.42)	26.25 (0.93)
		$\sigma_{A_{D}}^{2}$	4.39 (1.04)	3.68 (1.52)	4.23 (0.89)
		$\sigma_{c}^{2}$	2.55 (0.57)	5.32 (1.30)	3.00 (0.58)
		$h_{D}^{2}$	0.17 (0.04)	0.13 (0.05)	0.16 (0.03)
		$c^{2}$	0.10 (0.02)	0.19 (0.06)	0.11 (0.05)

^1^Estimates of $\sigma_{e}^{2}$ at birth was 0.43 (0.01).

^2^Estimates of $\sigma_{e}^{2}$ at weaning was 19.02 (0.58).

The direct and maternal additive correlations between purebred and crossbred breed types were in general moderately high to high. At birth, for the heterogeneous residual variance model, the direct additive correlation between Columbia and crossbred lambs was 0.58 ± 0.35, and between Suffolk and crossbred lambs was 0.85 ± 0.22. The maternal additive correlations between Columbia and crossbred lambs and between Suffolk and crossbred lambs were 0.71 ± 0.13 and 0.78 ± 0.16, respectively. For the homogeneous residual model, the direct additive correlation between Columbia and crossbred lambs was 0.68 ± 0.30 and between Suffolk and crossbred lambs was 0.90 ± 0.22. Similarly, maternal additive correlations were 0.75 ± 0.15 and 0.72 ± 0.16 between Columbia and crossbred lambs and between Suffolk and crossbred lambs, respectively.

At weaning, for the heterogeneous residual variance model, direct additive correlation between Columbia and crossbred lambs was 0.96 ± 0.21 and between Suffolk and crossbred lambs was 0.43 ± 0.38. For the homogeneous variance model, the direct additive correlation was 0.94 ± 0.29 between Columbia and crossbred lambs and 0.42 ± 0.40 between Suffolk and crossbred lambs.

### Validation

Including crossbred progeny in the evaluation of purebred Columbia and Suffolk sires resulted in increased accuracy of prediction of progeny performance. The average accuracy when only purebred progeny were included in the evaluation was 0.65 ± 0.01 and 0.69 ± 0.01 for Columbia and Suffolk sires, respectively. When both purebred and crossbred progeny were included in the evaluation, the average accuracy of Columbia and Suffolk sires was 0.67 ± 0.01 and 0.72 ± 0.01, respectively, representing an increase of 2.86% and 4.59%, respectively.

At birth, the confidence intervals for the slopes resulting from regressing adjusted progeny performance on sire EBV included the expected value of 0.50 in all scenarios of validation and evaluation (Table 4). However, regression coefficients were more accurately estimated (smaller SE and higher coefficients of determination) when the validation set combined birth weights on purebred and crossbred progeny, while the estimation set used to predict sire EBV used either purebred or combined birth weights. This advantage roughly corresponded to the larger number of sires and progeny available for validation.

**Table 4.** Number of sires, progeny, slope (standard error in parentheses), and coefficient of determination (*R*^*2*^) for a linear model^1^ fitted to progeny records from the validation set at birth.

Breed	Evaluation^2^	Validation^3^	Sires (*n*)	Progeny (*n*)	Slope	*R* ^2^
Columbia	Purebred	Purebred	114	2,876	0.57 (0.11)	0.24
		Crossbred	35	268	0.63 (0.17)	0.16
		Combined	115	3,144	0.46 (0.10)	0.25
	Combined	Purebred	81	2,502	0.47 (0.11)	0.24
		Crossbred	22	194	0.42 (0.19)	0.17
		Combined	84	2,696	0.48 (0.11)	0.28
Suffolk	Purebred	Purebred	96	1,588	0.53 (0.10)	0.25
		Crossbred	60	483	0.61 (0.17)	0.17
		Combined	96	2,072	0.45 (0.08)	0.26
	Combined	Purebred	30	1,272	0.48 (0.09)	0.31
		Crossbred	42	409	0.41 (0.18)	0.32
		Combined	68	1,681	0.49 (0.08)	0.27

^1^Linear model included birth weight as dependent variable, contemporary group, dam age, birth type, heterosis (for crossbred lambs), sire EBV (as a continuous covariate), and residual.

^2^Evaluation set: genetic evaluations for purebred Columbia and Suffolk sires using best linear unbiased prediction based on either purebred progeny only or combined purebred and crossbred progeny.

^3^Validation set: progeny performance records used to obtain regression coefficient and coefficient of determination in a linear model.

At weaning, the confidence interval for regression coefficients of sire EBV on adjusted progeny performance also included 0.5 in all cases (Table 5). However, due to the reduced number of records available for validation, higher standard errors were found generally. As with birth weight, overall higher coefficients of determination were found when sire EBV was obtained as part of a combined purebred and crossbred evaluation.

**Table 5.** Number of sires, progeny, slope (standard error in parentheses), and coefficient of determination (*R*^*2*^) for a linear model^1^ fitted to progeny records from the validation set at weaning.

Breed	Evaluation^2^	Validation^3^	Sires (*n*)	Progeny (*n*)	Slope	*R* ^2^
Columbia	Purebred	Purebred	105	1,733	0.52 (0.19)	0.22
		Crossbred	33	202	0.65 (0.22)	0.14
		Combined	105	1,936	0.45 (0.17)	0.18
	Combined	Purebred	106	1,735	0.43 (0.16)	0.21
		Crossbred	34	203	0.35 (0.19)	0.18
		Combined	114	3,042	0.55 (0.10)	0.26
Suffolk	Purebred	Purebred	77	650	0.53 (0.19)	0.21
		Crossbred	60	352	0.67 (0.21)	0.15
		Combined	83	1,002	0.47 (0.16)	0.19
	Combined	Purebred	55	463	0.58 (0.17)	0.23
		Crossbred	58	366	0.45 (0.20)	0.16
		Combined	66	829	0.52 (0.15)	0.26

^1^Linear model included birth weight as dependent variable, contemporary group, dam age, birth type, heterosis (for crossbred lambs), sire EBV (as a continuous covariate), and residual.

^2^Evaluation set: genetic evaluations for purebred Columbia and Suffolk sires using best linear unbiased prediction based on either purebred progeny only or combined purebred and crossbred progeny.

^3^Validation set: progeny performance records used to obtain regression coefficient and coefficient of determination in a linear model.

At birth and weaning, for both Columbia and Suffolk sires, moderate under-dispersion for sire EBV (regression coefficient values ≥ 0.5) was observed when predicting purebred (only) and crossbred (only) progeny performance from sire EBV based on a purebred progeny only evaluation. On the other hand, at birth slight over-dispersion (values ≤ 0.5) was observed when predicting purebred, crossbred, and combined progeny performance based on sire EBV obtained from a combined evaluation. Such was not the case at weaning, where both under- and over-dispersion was observed for the combined evaluation.

### Purebred sire EBV

**Figure 6.**
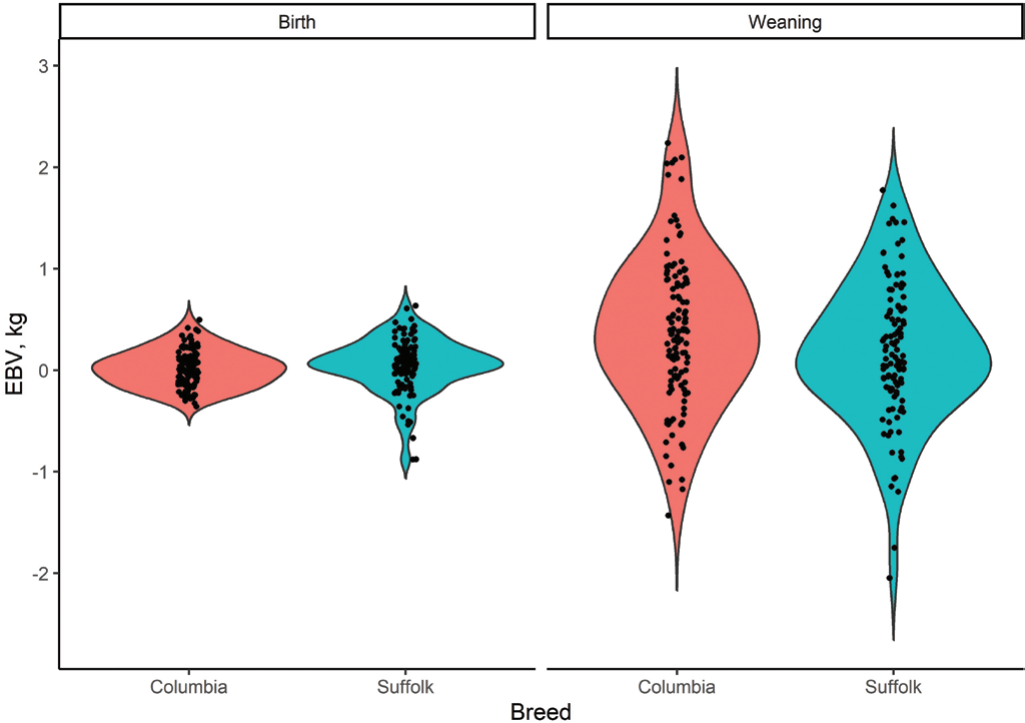
Distribution of EBV (shown as black circles) for Columbia and Suffolk sires at birth and weaning resulting from a combined (multiple-breed) evaluation including crossbred progeny.

Columbia and Suffolk sire EBV for birth and weaning weights resulting from a multiple-breed evaluation which included crossbred progeny are presented in Figure 6.

While the distribution of sire EBV was fairly symmetric at both stages of growth, at birth and weaning, negative values for Suffolk sire EBV resulted in a slightly left-skewed distribution compared to those of Columbia sires. In both cases, this could be a consequence of the relatively limited number of sires evaluated. The presence of extreme values would likely be less pervasive in a larger sample of sires (i.e., national evaluation). Regardless, there was considerable overlap in the distribution of EBV for both birth and weaning weight in the two breeds.

## Discussion

### Heterosis estimates

Estimates of direct and maternal heterosis were larger than those reported elsewhere (Brown et al., 2016; Vargas Jurado et al., 2022) with less differences with the latter study. The discrepancy with Brown et al. (2016) could be due to the breeds used in their study (Poll Dorset, Suffolk, Texel, and White Suffolk). Moreover, in the present study, records from the purebred and crossbred lambs were considered different traits, which was not the case in Brown et al. (2016). In addition, the dataset used in the current study contained reciprocal crosses, mainly Columbia-Suffolk and Columbia-Suffolk-Texel, such that effects were perhaps better estimated compared to other studies. Also, because Columbia and Suffolk (and Texel) belong to different breed types, heterosis effects were likely more pronounced. Given the overlap in the type of breeds used in our current study and Vargas Jurado et al. (2022), similar results were anticipated. Nevertheless, the magnitude of the estimates of heterosis highlights the benefit of using crossbreeding to improve productivity in sheep.

### Connectedness among and within contemporary groups

Estimates of connectedness (relatedness) showed that the purebreds were strongly linked genetically (connectedness correlations above 0.50) with crossbred breed types. These links were due to crossbred progeny of purebred sires having performance records in the same contemporary group.

The estimates of variance components (genetic correlations) between purebred and crossbred animals, therefore, could be reasonably estimated. Márquez et al. (2015) reported that strong connectedness was achieved between Suffolk, Charollais, and Texel sires—connectedness correlations of 0.40—through their crossbred progeny reared contemporaneously on three farms. However, the weaker relatedness we observed between the Columbia and Suffolk breeds at birth and weaning reinforced our setting the direct and, for birth weight, maternal additive (co)variance between breeds to zero.

For BLUP to effectively disentangle environmental effects from those due to additive effects, genetic linkages among farms and environments are necessary. This is particularly the case in commercial settings where crossbred progeny may be raised in a different environment than that of purebreds. As such, for a practical implementation of a genetic evaluation including crossbred information, the sufficiency of those linkages needs to be assessed beforehand to ensure breed-effects are estimable and to reduce bias in the EBV (Kuehn et al., 2007, 2008; Márquez et al., 2015). In our study, purebred and crossbred lambs were raised in similar conditions, although some aspects of ewe management differed among breed types. Still, our definition of contemporary groups, and the relatively strong genetic linkages among breed types, ensured that direct comparisons among purebred and crossbred lambs were robust.

### Dam age

At birth, the heaviest lambs were produced by 6-yr-old ewes regardless of breed type. The mean difference between lambs born from 1-yr-old and 2-yr-old ewes was 0.85, 1.26, and 1.54 kg for Columbia, Suffolk, and crossbred lambs, respectively. Stobart et al. (1986) reported a difference of 0.50 kg between lambs born from 2-yr-old and mature (4 + yr) western-range ewes. Pettigrew et al. (2019) reported slightly larger differences (0.80 kg) in the birth weight of lambs born from ewe-lambs compared to mature dual purpose Romney ewes. Similarly, Fahmy (1982) reported a mean difference of 0.81 kg between litters born from 3- and 4-yr-old ewes versus litters born from 2-yr-old ewes for Suffolk and Oxford sired lambs.

At weaning, the mean difference between lambs born from 1-yr-old and 2-yr-old ewes was 5.23, 5.81, and 6.12 kg for Columbia, Suffolk, and crossbred lambs, respectively. Similarly, Pettigrew et al. (2019) reported a difference of 6.00 kg for lambs born from young ewes compared to those born from mature Romney ewes. On the other hand, Stobart et al. (1986) reported a mean difference of 2.10 kg at weaning, respectively, between lambs born from 2-yr-old ewes and lambs born from mature western-range ewes.

In our study, the predicted birth weight values for a given dam age in crossbred lambs were substantially larger than the purebred and the parental breed average. At weaning, however, predicted body weight values for a given dam age for crossbred lambs more closely resembled the predicted Suffolk dam age effect. Moreover, differences among breed types were less clear than at birth. Still, the predicted weight for Columbia lambs was, in general, lower than Suffolk and crossbred lambs, perhaps reflecting a slower maturing rate in the Columbia breed (Leymaster and Smith, 1981; Snowder et al., 1994). Given the difference in the predicted effect of dam age for purebreds and crossbreds, especially at birth, different adjustment factors would likely be required when evaluating crossbred progeny.

### Birth (and rearing) type

At birth, lambs born as singles were on average 1.47, 1.64, and 1.36 kg heavier compared to lambs born as twins+ for the Columbia, Suffolk, and crossbred breed types, respectively. McHugh et al. (2017) reported differences of about 1.03 kg for lambs born as singles compared to those born as twins (or triplets). The differences observed in our study, however, were considerably larger than those reported by Pettigrew et al. (2019), Stobart et al. (1986), and Notter and Brown (2015) who reported a 0.70 and 0.97 kg difference, respectively, between lambs born as single and lambs born as twins.

At weaning, lambs raised as singles were 5.35, 5.47, and 5.91 heavier than lambs born and raised as twins+ for the Columbia, Suffolk, and crossbred breed types, respectively. Stobart et al. (1986) found differences of 1.90 kg between lambs born and raised as singles and lambs born as twins but raised as singles, and differences of 5.40 kg between lambs born and raised as singles and lambs born and raised as twins. Similarly, Pettigrew et al. (2019) and Notter and Brown (2015) reported a body weight difference of 5.50 and 4.68 kg, respectively, for lambs born as singles compared to lambs born as twins.

While the effect of birth (or birth-rearing) on body weight at birth and weaning was clear, differences among purebred and crossbred lambs in the pattern of these changes were less clear. Similar effects were reported by Notter and Brown (2015). At birth, the predicted effect of birth type for crossbreds was smaller compared to those of purebred lambs. At weaning such was not the case. Notter and Brown (2015) highlighted the need for the use of adjustment factors derived from exponential functions to decrease bias in EBV estimation in multi-breed evaluations. Such adjustments should also be considered for the evaluation of crossbred progeny, especially at birth when differences among breeds are more evident.

### Variance components

Based on both the log-likelihood ratio and Bartlett’s tests, the additive variances (direct and maternal) differed among breed types at birth and weaning. On the other hand, there was a discrepancy between the tests regarding the residual variance. This may be due to Bartlett’s test considering only point estimates of variances and the number of records for each breed type, while the log-likelihood ratio test is a function of the phenotypic records, fixed and random effects, and variance components. As such, small changes in some variance components (e.g., residual variance) deemed significant by Bartlett’s test may not be large enough to be considered significant by the likelihood ratio test. Still, ratios of variance components in both the homogeneous and heterogeneous case where very similar (within the confidence limits).

Márquez et al. (2015) reported a better fit for the evaluation of crossbred lambs sired by Suffolk, Texel, or Charollais rams when the model included heterogeneous variances. Similarly, Tosh and Kemp (1994) suggested the use of heterogeneous variances when using across-breeds evaluations. In the present study, both phenotypic and residual variances differed between purebred and crossbred lambs at birth and weaning. At birth, the residual variance for the crossbreds was larger than those of Columbia and Suffolk; at weaning, the residual variance was smaller. As such, in a multi-breed evaluation, a model accounting for heterogeneous residual variances may be more appropriate when records from crossbred progeny are included.

Estimates of phenotypic variances at birth were similar to those reported by Borg et al. (2009) for Columbia sheep and those reported by Vargas Jurado et al. (2022) for multiple breeds. Similarly, estimates of direct and maternal heritability were close to those reported elsewhere (Brown et al., 2016; Vargas Jurado et al., 2022). The proportion of variance explained by the maternal additive component emphasizes the importance of including maternal effects when modeling growth traits, especially pre-weaning (Vargas Jurado et al., 2022). Estimates of the correlation for direct additive effects between Suffolk and crossbred lambs at birth, and between Columbia and crossbred lambs at weaning, were consistent with those reported by Brown and van der Werf (2015) for Dorset sheep and their crosses. Given the magnitude of direct and maternal genetic correlations, including crossbred information could benefit the evaluation of purebreds by increasing their accuracy.

### Validation

The coefficients from the regression of adjusted progeny performance (body weight) on sire EBV for weaning weight were more variable (i.e., larger standard errors) than those reported by Huisman et al. (2016) for untransformed (observed scale) weaning weight measured in Suffolk, White Suffolk, Poll Dorset, and Texel sheep. Such was also the case for comparable regression analyses of weaning weight in Poll Dorset and White Suffolk sheep reported by Paneru et al. (2022). In Huisman et al. (2016) and Paneru et al. (2022) regression coefficients were also close to the expected value of 0.50. Given the larger size of those studies, sire EBV and adjusted progeny performance would be expected to be more accurately estimated, resulting in lower variability as compared to our study.

For Columbia and Suffolk sires at birth, the *R*^*2*^ associated with the prediction of purebred progeny performance was unchanged when the performance of crossbred progeny was either included or excluded from a purebred evaluation. On the other hand, a combined evaluation, including crossbred progeny records improved predictions for both crossbred or combined (purebred and crossbred) progeny performance (based on *R*^*2*^ values). Furthermore, because *R*^*2*^ did not decrease in any of the cases, including crossbred progeny records would not deteriorate predictions of purebred progeny performance.

At weaning, including crossbred progeny records for the prediction of purebred Columbia sires resulted in a small decrease in *R*^*2*^. Such was not the case for Suffolk sires, where including crossbred performance increased *R*^*2*^ values in all cases. However, the increase was less substantial than at birth. The decrease in fit for Columbia sires, although very minor, may be due to the reduction in the number of records and the number of sires available at weaning compared to at birth. Still, as for birth weight, confidence intervals for regression coefficients included 0.50 in all scenarios, suggesting that predictions of purebred, crossbred, or both, based on a combined evaluation would be reliable. The increases in accuracy of EBV at both birth and weaning or, conversely, the decreases in the standard errors of sire EBV, can be explained by the increase in the number of progeny records for a given sire. The additional information garnered through genetic linkages among breeds also contributed to the overall increase in reliability of sire EBV, although likely to a lesser extent than the increase in progeny numbers.

Paneru et al. (2022) reported over-dispersion of sire EBV at weaning, the extent of which depended on the model definition used to calculate the adjusted progeny performance and adjustment factors. Moreover, Huisman et al. (2016) reported under-dispersion when predicting progeny performance at weaning in medium and high-production environments with over-dispersion in low-producing environments. Also, those authors showed that several transformations applied to weight measurements to approximate normality had a varied impact on the value of the regression coefficients. In our study, the patterns of under- and over-dispersion were not clear but could potentially be alleviated if more comprehensive models (permanent and temporary maternal environmental effects, maternal genetic group effects) could have been fitted. As such, these factors should also be considered in future analyses that include crossbred and purebred progeny to reduce potential biases in sire EBV.

### Purebred sire EBV

In the current study, it was possible to include performance records from crossbred progeny into a multiple-breed evaluation of purebred Columbia and Suffolk rams because crossbred progeny belonged to the same contemporary (management) group. This highlights the importance of crossbreeding strategies that allow for sufficient genetic connections among the different breeds evaluated. Because of these connections, the resulting sire EBV was on the same scale and thus could be directly comparable. This is an additional benefit of the current model.

While comparing sires of breed types with different purposes (e.g., maternal and terminal, hair, and wool) may be of less interest to commercial producers in the U.S., the possibility of selecting rams across breeds being used for similar purposes (e.g., within terminal or maternal breed types) provides breeders with an additional tool when making selection decisions, as well as the potential for higher genetic gains due to increased genetic variability. The ability to directly compare Columbia and Suffolk rams for growth traits fits such a scenario since western-range producers consider the relative benefits of using Columbia versus Suffolk rams as terminal sires to produce market lambs. The large overlap in EBV for birth and weaning weight observed in this pair of breeds suggests that producers have considerable flexibility in their decision-making. Furthermore, such across-breed evaluations are already available to Australian sheep (Brown et al., 2007, 2016) and to beef cattle (Kuehn and Thallman, 2017) breeders.

## Conclusions

Age of dam and birth and rearing type had strong effects on birth weight with distinct patterns of change in purebred and crossbred breed types. Such differences among breed types, however, were less clear at weaning. These results may be useful for delineating management practices during gestation for purebred and crossbred ewes depending on their age and pregnancy status to mitigate the effect of dam age on birth weight. Also, to accurately compare breeds with little biases in a joint genetic evaluation, beyond adjustments due to heterotic effects, accounting for dam age and birth type differential by breed type may be necessary for birth weight.

Evaluation of purebred animals could benefit from additional information from crossbred lambs due to moderate to strong genetic correlations among purebreds and crossbreds. However, the extent of this benefit would depend on the realized linkages (connectedness) among sires and dams of different breeds in the evaluation (across flocks).

This study constitutes an additional step towards implementing genetic evaluation including crossbred information in the U.S. sheep industry by identifying factors, namely differing impacts of systematic effects and heterogeneous variances, necessary to consider for reliable prediction. Beyond those considerations, the actual changes in sire EBV and their accuracy when including crossbred progeny in a large-scale genetic evaluation need to be tested before commercial implementation of such a scheme, all of which are the next steps in our research.

## Abbreviations

BLUP: best linear unbiased prediction
EBV: estimated breeding values
PC: paternal composite line
TS1: first terminal-sire evaluation project
TS2: second terminal-sire evaluation project
USSES: U.S. Sheep Experiment Station
